# Critical evaluation and recalculation of current systematic reviews with meta-analysis on the effects of acute and chronic stretching on passive properties and passive peak torque

**DOI:** 10.1007/s00421-024-05564-6

**Published:** 2024-07-27

**Authors:** Konstantin Warneke, Lars Hubertus Lohmann, Gerit Plöschberger, Andreas Konrad

**Affiliations:** 1 Institute of Human Movement Science, Sport and Health, Karl-Franzens University of Graz, Mozartgasse 14, 8010 Graz, Austria; 2 Institute of Sport Science, University of Klagenfurt, Klagenfurt am Wörthersee, Austria; 3 Department of Human Motion Science and Exercise Physiology, University of Jena, Jena, Germany

**Keywords:** Muscle stiffness, Tendon stiffness, Passive resistance, Stretching pain, Muscle–tendon unit, Pain threshold

## Abstract

**Purpose:**

Muscle, tendon, and muscle–tendon unit (MTU) stiffness as well as passive peak torque (PPT) or delayed stretching pain sensation are typical explanatory approaches for stretching adaptations. However, in literature, differences in the study inclusion, as well as applying meta-analytical models without accounting for intrastudy dependency of multiple and heteroscedasticity of data bias the current evidence. Furthermore, most of the recent analyses neglected to investigate PPT adaptations and further moderators.

**Methods:**

The presented review used the recommended meta-analytical calculation method to investigate the effects of stretching on stiffness as well as on passive torque parameters using subgroup analyses for stretching types, stretching duration, and supervision.

**Results:**

Chronic stretching reduced muscle stiffness ( −  0.38, *p* = 0.01) overall, and also for the supervised ( −  0.49, *p* = 0.004) and long static stretching interventions ( −  0.61, *p* < 0.001), while the unsupervised and short duration subgroups did not reach the level of significance (*p* = 0.21, 0.29). No effects were observed for tendon stiffness or for subgroups (e.g., long-stretching durations). Chronic PPT (0.55, *p* = 0.005) in end ROM increased. Only long-stretching durations sufficiently decreased muscle stiffness acutely. No effects could be observed for acute PPT.

**Conclusion:**

While partially in accordance with previous literature, the results underline the relevance of long-stretching durations when inducing changes in passive properties. Only four acute PPT in end ROM studies were eligible, while a large number were excluded as they provided mathematical models and/or lacked control conditions, calling for further randomized controlled trials on acute PPT effects.

**Supplementary Information:**

The online version contains supplementary material available at 10.1007/s00421-024-05564-6.

## Introduction

In the literature, there is an indisputable agreement about the beneficial acute and chronic effects of stretching on range of motion (ROM) (Arntz et al. 2023; Behm et al. 2023; Konrad et al. 2023; Warneke et al. 2024a, b, c). Due to its known injury-preventive influence, stretching has been an integral component of movement preparation routines for a long time (Behm et al. 2016, 2021a, b; Morrin and Redding 2013; Worrel et al. 1994). In addition to the inclusion in warm-up routines, stretching is often considered in athletic and therapy programs with a focus on improving or restoring muscle extensibility, e.g., after prolonged periods of immobilization due to injury (Warneke et al. 2022a, b; Wilson et al. 2019) or to treat muscular imbalances (González-Gálvez et al. 2019) or back pain (Fontana Carvalho et al. 2020; Zhu et al. 2020).

Even though stretching is frequently performed in practice, the underlying mechanisms and biologic adaptations of the muscles and the surrounding tissue that contribute to enhanced ROM and other benefits are a matter of ongoing debate. While Reiner et al. (2024) and Konrad et al. (2024) indicated a relationship between flexibility and stiffness, previous studies have suggested that sustained changes in muscle morphology as an acute response to a single stretching bout are unlikely (Behm et al. 2016; Opplert et al. 2016). Accordingly, in 2018, Freitas et al. reviewed the available literature on long-term stretching adaptations and reported no meaningful chronic reductions in stiffness-related parameters in the muscle, the tendon, and the muscle–tendon unit (MTU) following up to 8-week stretching protocols that incorporated a weekly stretching volume of up to 20 min. Accordingly, irrespective of acute or chronic stretching, flexibility effects have been mainly attributed to neuromuscular adaptations such as increased stretch or pain tolerance, rather than changes in the muscle–tendon structure (Freitas et al. 2018; Konrad and Tilp 2014).

However, most recently, paradigms have changed. In previous meta-analyses, Takeuchi and colleagues summarized the acute-stiffness reduction of the MTU (effect size (ES) =   −  0.772, *p* = 0.018) (Takeuchi et al. 2023a, b). They also suggested that chronic stretch training can influence muscle stiffness parameters when using durations of up to 6 × 5 min of stretch (Nakamura et al. 2021) for up to 12 weeks (Longo et al. 2021; Takeuchi et al. 2023a). However, although providing interesting results, the two recent analyses by Takeuchi et al. (2023a, b) did not include passive torque parameters, which have been previously described as a factor of interest that could provide further explanations for the result discrepancies between the Takeuchi et al. (Takeuchi et al. 2023a, b) and Freitas et al. (2018) reviews. These discrepancies could be attributed to, on the one hand, emerging evidence (5 vs. 10 papers) within the last 6 years (search performed in 2016 vs. 2022) and, on the other hand, differences in the applied meta-analytical calculation models. In the literature, two different approaches to handling multiple effect sizes (ES) are commonly described: (1) multilevel meta-analyses; and (2) averaging the ES, which, unfortunately, can result in information loss and/or overestimated ES (Fisher and Tipton 2015). While Freitas et al. (2018) did not report any of the two approaches to handle multiple within-study outcomes, Takeuchi et al. (2023a, b) used the within-study mean (second approach) if more than one effect size was available from one study. To counteract result biasing from applying inappropriate effect size pooling and account for within-study dependencies and the unknown covariance structure of multiple outcomes, Fisher and Tipton (2015) recommended using the robust variance estimation model (RVE).

Consequently, we reviewed the available acute and chronic literature to evaluate the stretch-induced adaptations in PPT parameters and muscle, tendon, and MTU-stiffness parameters by using the recommended calculation method. Furthermore, the results are discussed based on several moderator variables to provide a deeper insight into the explanation of ROM increases via underlying physiologic mechanisms.

## Methods

Adhering to the Preferred Reporting Items for Systematic Reviews and Meta-Analyses (PRISMA) guidelines, we screened the literature to provide a replicable search, while using RVE meta-analytical effect size pooling for the effect quantification. Ethical publishing standards were considered (Wager & Wiffen 2011) and the study was registered in the PROSPERO database (ID:CRD42023451509).

### Literature search

Considering the Population, Intervention, Comparison, and Outcome (PICO) guidelines, two independent authors (KW and LHL) conducted a systematic literature search using MEDLINE/PubMed and Web of Science (inception to November 2023), which was supplemented by a manual search using the first 500 hits from Google Scholar. The following criteria were applied for study inclusion:

1. healthy human participants of all ages;
2. a controlled intervention study;
3. studies exploring acute and/or chronic responses;
4. measurement of muscle stiffness, tendon stiffness, muscle–tendon unit, stiffness, passive resistive torque, or passive peak torque, which were measured via isokinetic dynamometry or ultrasound investigation.

Trials were not considered in this review if they:

- combined different interventions (e.g., stretching plus strengthening);
- included patients (such as multiple sclerosis patients); or
- used strain elastography.

Thus, the search terms were built adhering to the PICO guidelines. The search terms were created based on the requirements of each database. The literature search in PubMed was conducted using the following terms:

(“passive torque” OR “passive propert*” OR “resistive torque” OR “pain perception” OR “muscle stiffness” OR “tendon stiffness” OR “muscle–tendon stiffness” OR “MTU” OR “stretch tolerance” OR “joint stiffness” OR “muscle tendon unit stiffness” OR “elastic modulus” OR “shear modulus” OR “passive tension”) AND (“control*” OR “cross-over” OR “crossover”) AND (“stretch*”) NOT (“patients” OR “animal*”).

In addition, the reference lists of the included studies, as well as the related reviews, were screened for further eligible articles (Horsley et al. 2011). Chronic stretching interventions were considered if at least one stretching session per week for a minimum of 2 weeks was performed. The intervention was considered static stretching if it involved lengthening of a muscle until stretch sensation/the point of discomfort (POD) which was held in a lengthened position for a prescribed duration (Behm 2018). Proprioceptive neuromuscular facilitation (PNF) stretching added a maximal voluntary contraction to a static stretch with or without antagonist contraction (CR or CRAC) (Behm 2018). In accordance with Warneke et al. (2024a, b, c), dynamic stretching was defined as controlled back and forth movements in the end ROM with ballistic stretching as a subcategory that included less controlled and bouncing movements in the end ROM.

### Methodological study quality and risk of bias

Risk of bias was evaluated via the PEDro scale for the assessment of the methodological quality of clinical trials (de Morton 2009; Maher et al. 2003). Scoring was performed by two independent investigators (KW and LHL), with interrater reliability expressed as Intraclass Correlation Coefficients for agreement of 0.992. If a consensus was not reached, a third examiner provided the decisive vote (GP). The risk of bias assessment was supplemented by examining the risk of publication bias by visually inspecting modified funnel plots (2020) to account for multiple within-study outcomes. Egger’s regression test with the extension for dependent ES (Fernández-Castilla et al. 2020) supplemented the visual inspection.

Furthermore, the certainty of the evidence was assessed by applying the Grading of Recommendations, Assessment, Development, and Evaluations (GRADE) Working Group criteria (Atkins et al. 2004), considering the following categorization: “very low” to rate the estimate of the effects as very uncertain; “low”, indicating that further research would very likely change the effect estimation; “moderate” with a very likely change of effect estimation; and “high”, indicating further research to be very unlikely to change the estimated effect. Following the initial categorization of high, moderate or low — based on the design of included studies — the quality of the evidence is consequently adjusted, based on different criteria, e.g., study design, inconsistency, directness, imprecision, and risk of bias, with one point subtracted for each weakness. On the contrary, large magnitude effects or a dose–response gradient led to improvement of the quality of evidence by one point each.

### Data processing and statistics

First, the mean (M) and standard deviation (SD) values from the pre-tests and post-tests for all parameters were collected to calculate the changes from pre-test to post-test with $M_{{{\text{Diff}}}} = M_{{{\text{post}}}} - M_{{{\text{pre}}}}$. SDs were pooled as $SD_{{{\text{pooled}}}} = \sqrt {\frac{{\left( {n_{1} - 1} \right)*SD_{1}^{2} + \left( {n_{2} - 1} \right)*SD_{2}^{2} }}{{\left( {n_{1} - 1} \right) + \left( {n_{2} - 1} \right)}}}$.

In the case of missing data, the authors of the primary studies were contacted via E-Mail or ResearchGate. If neither pre-and post-values nor SDs were provided, and these could not be imputed from graphics, the study was excluded from further processing.

A meta-analysis with RVE was used to account for the dependency of the ES (i.e., in the case of multiple outcomes in the same study). The standardized mean differences (SMDs) with 95% confidence intervals (CIs) between the commonly used stretching interventions (static and dynamic stretching or PNF) and control group were calculated and are provided in the result tables. In addition to the omnibus analyses on the effects of stretching on passive properties in general, subgroup analyses were performed for the different types of stretching (static, dynamic, PNF), including passive torque measurements with (isokinetic) dynamometry, while muscle and tendon stiffness was assessed via B-mode ultrasound technology, and MTU stiffness was assessed in combination with dynamometry. Other measurement techniques (e.g., strain elastography or calculation models) were excluded to increase the homogeneity and comparability of the results. To assess the influence of the stretching duration in static stretching subgroup analyses were performed. Since no consensus exist when stretching duration with regard to stretching can be considered long or short, the included stretching times were median split (long vs. short) for stretching volume per session (means that if 4 × 30s was applied, 120s was used for further calculation). The remaining cut offs are provided in the Tables 3 and 4, if subgroup separation was performed. In addition, the effect of supervision was also explored via subgroup analyses. The between-study variance component was estimated using τ^2^. Pooled ES were interpreted as follows: 0 ≤ ES < 0.2 trivial, 0.2 ≤ ES < 0.5 small, 0.5 ≤ ES < 0.8 moderate, and ES ≥ 0.8 large (Faraone 2008). All the calculations were performed using R and the robumeta package (Fisher and Tipton 2015).

## Results

### Search results and study characteristics

**Fig. 1:**
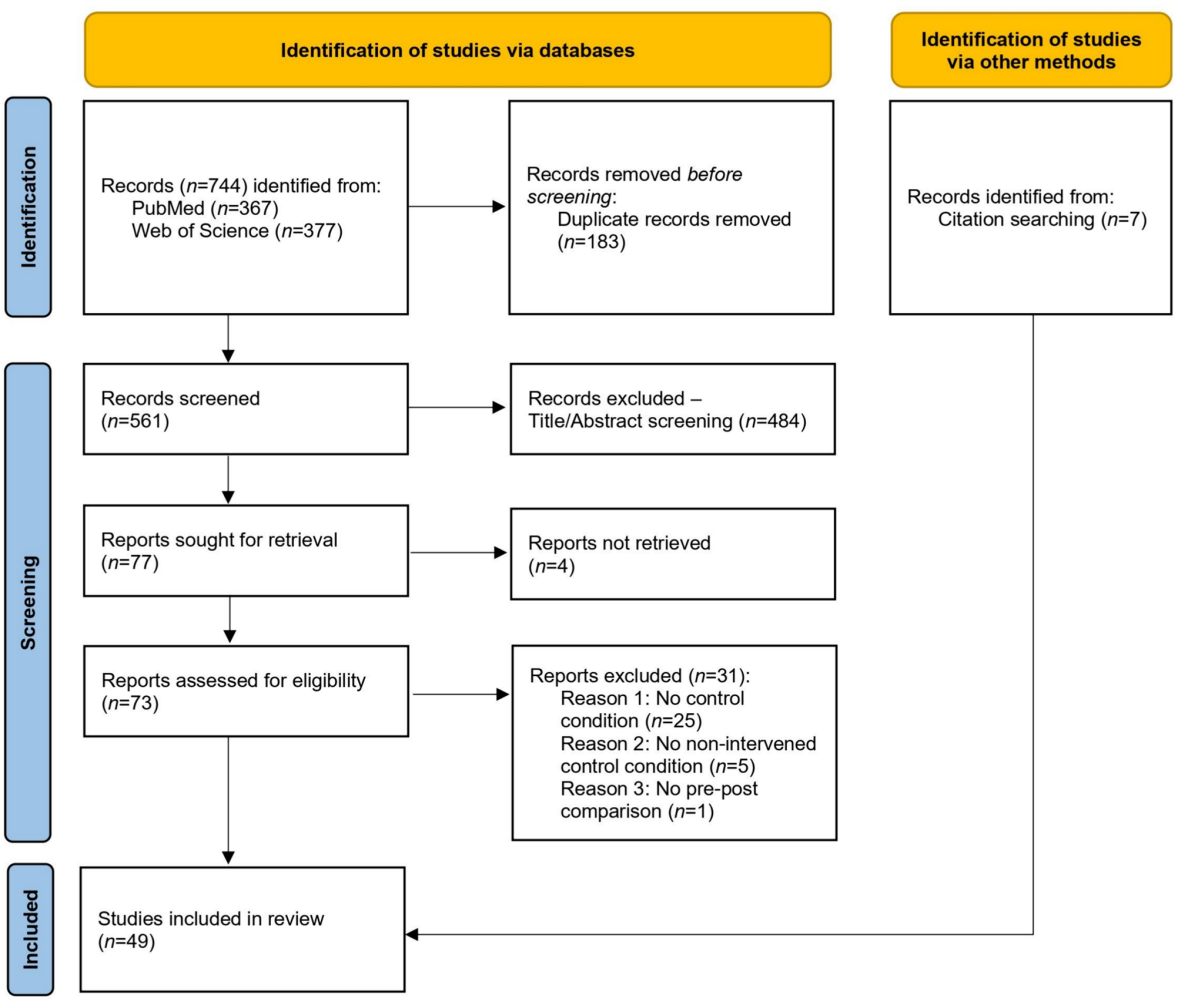
PRISMA flow chart to illustrate the systematic literature search

Figure 1 illustrates the literature search, showing a total of 49 included studies (*n* = 1304 participants), while the acute and chronic study characteristics are provided in Table 1 (chronic) and Table 2 (acute). Thirty of the papers examined the chronic effects of static stretching, while 19 studies investigated the acute effects of static stretching. With 48 studies, the majority of the articles addressed muscles of the lower extremity, while only one study was included targeting upper body muscles (Reiner et al. 2023). Twenty-nine studies supervised the intervention, while ten each reported either self-stretching or did not report information. Chronic stiffness was investigated in 18 studies, with 14 investigating muscle stiffness and seven studies including tendon stiffness. PPT was measured in end ROM in ten studies, and 11 studies measured PPT at a fixed angle in pre-post comparisons.

**Table 1:** Study characteristics of the included chronic studies

Study	Participants	Intervention	Material and testing	Outcome
Akagi and Takahashi, (2014)	*n* = 19 healthy men (intra-individual control leg), 23.7 ± 2.3 yrs. of age. 6 participants were sedentary and the others engaged in recreational sport 1-8h/wk Randomized allocation of control/intervention leg	Self-administered static stretching. Intervention period: 5 wks. Frequency: 6x/wk. Duration per session: 3 × 120s with a 60s rest between bouts Muscle group: Plantar flexors	Isokinetic dynamometer. Ultrasound with shear wave elastography at 11 Hz	MTU stiffness (Nm/°)
Andrade et al. (2020)	*n* = 60 healthy participants (IG1-MDS: n = 21. IG2-NDS: n = 21. CG: n = 18. F = 29. M = 31), 20.5 ± 2 yrs. of age. Physically active (all participants reported weekly physical activity > 8h) Randomized group allocation Post-test: IG2-NDS: *n* = 20. CG: *n* = 17	Supervised nerve- or muscle-directed sciatic stretching. Intervention period: 12 wks. Frequency: 5x/wk. Duration per session: 5 × 45s with < 5s rest between bouts Muscle groups: Hamstrings & plantar flexors	Dynamometer. B-mode ultrasound (50 mm, 4–15 MHz linear transducer for gastrocnemius and 38 mm, 2–10 MHz linear transducer for soleus and sciatic nerve) with shear wave elastography	Shear wave velocity, passive torque (Nm)
Ben and Harvey (2010)	*n* = 60 healthy participants (IG: *n* = 30, M = 9, F = 21, 35 ± 12 yrs. of age. CG: *n* = 30, M = 7, F = 23, 39 ± 12 yrs. of age) Randomized group allocation	Supervised static stretching (18/30 stretch interventions supervised). Intervention period: 6wks. Frequency: 5x/wk. Duration per session: 1800s Muscle group: Hamstrings	Wheel with attached leg splint with weights hanging from the wheel rim	Highest tolerated torque (Nm), Standardized torque (muscle extensibility)
Blazevich et al. (2014)	*n* = 22 healthy men (IG: *n* = 12. CG: *n* = 10), 18.6 ± 0.9 yrs. of age Randomized group allocation Post-test: CG: *n* = 9	Self-administered static stretching. Intervention period: 3 wks. Frequency: 14x/wk. Duration per session: 4 × 30s with 15s rest between bouts Muscle group: Plantar flexors	Dynamometer. B-mode ultrasound (39 mm, 8 MHz linear transducer) at 29 Hz	Passive peak joint moment at end ROM (Nm), MTU stiffness (Nm/°)
Brusco et al. (2019)	*n* = 13 healthy men (intra-individual control leg), 23.6 ± 3.9 yrs. of age. All participants with limited hamstring flexibility. Participants were not engaged in sports programs No randomized allocation of control and intervention leg	Supervised static stretching. Intervention period: 6 wks. Frequency: 2x/wk. Duration per session: 8 × 60s with 30s rest between bouts Muscle group: Hamstrings	Isokinetic dynamometer	Relative passive torque (Nm), peak passive torque (Nm), maximum passive stiffness (Nm/°), relative passive stiffness (Nm/°)
Chan et al. (2001)	*n* = 40 healthy participants (IG1, IG2, CG1, CG2 each consisting of *n* = 10, M = 6, F = 4), 20 ± 3. yrs. of age Randomized group allocation (No information on sports background.)	Supervised static stretching. Intervention period: 4wks for IG 2, 8wks for IG1. Frequency: 3x/wk. Duration: 5 × 30s Muscle group: Hamstrings	Isokinetic dynamometer	Passive resistance (Nm)
Cini et al. (2023)	*n* = 30 healthy participants (IG1: *n* = 10, F = 9, M = 1. IG2: *n* = 10, F = 8, M = 2. CG: *n* = 10, F = 7, M = 3), 23.8 ± 4.4 yrs. of age. Physically active but not engaged in strength and flexibility training Randomized group allocation	Supervised static stretching. Intervention period: 6wks Frequency: 3x/wk. Duration: IG1 = 120s, IG2 = 300s Muscle group: Plantar flexors	Isokinetic dynamometer	Passive torque (Nm)
Gajdosik et al. (2005)	*n* = 19 aged women (IG: *n* = 10. CG: *n* = 9), 65–89 yrs. of age, with limited dorsiflexion range of motion. Minimally to moderately active Randomized group allocation	Self-administered static stretch. Intervention period: 8 wks. Frequency: 3x/wk. Duration per session: 10 × 15s Muscle group: Plantar flexors	Isokinetic dynamometer	Maximal passive dorsiflexion force (N), mean passive resistive force (N), average passive elastic stiffness (N/°)
Gajdosik et al. (2007)	*n* = 12 women (IG: *n* = 6. CG: *n* = 6), 18–31 yrs. of age. Minimally active and not participating in any exercise program Randomized group allocation Post-test: CG: *n* = 4	Static stretching. Intervention periods: 6 wks. Frequency: 5x/wk. Duration per session: 10 × 15s Muscle group: Plantar flexors	Isokinetic dynamometer	Passive torque (Nm), MTU stiffness (Nm/°)
Halbertsma and Göeken (1994)	*n* = 18 healthy adults (IG: *n* = 9. CG: *n* = 9. F = 10. M = 8), 26.5 yrs. of age (IG: 23.3. CG: 31.3). All participants with limited hamstring flexibility Randomized group allocation Post-test: IG: *n* = 7. CG: n = 7	Static stretching. Intervention periods: 4 wks. Frequency: 14x/wk. Duration per session: 1 × 600s Muscle group: Hamstrings	Electrically powered lift-frame	Maximum exerted passive muscle moment (Nm)
Ichihashi et al. (2016)	*n* = 30 healthy men (IG: *n* = 15. CG: *n* = 15), 22.7 ± 2.2 yrs. of age. All participants were non-athletes Randomized group allocation	Supervised static stretching. Intervention period: 4 wks. Frequency: 3x/wk. Duration per session: 1 × 300s Muscle group: Hamstrings	B-mode ultrasound shear wave elastography (50 mm linear transducer)	Muscle shear elastic modulus
Kay et al. (2018)	*n* = 26 healthy participants (IG: *n* = 13. CG: *n* = 13. F = 10. M = 16), 27.8 ± 8 yrs. of age. Recreationally active Randomized group allocation	Supervised dynamic stretching. Intervention period: 6 wks. Frequency: 2x/wk. Duration per session: 5 × 36s (3s stretch × 12 repetitions) with a 1s rest between repetitions and 60s rest between bouts Muscle group: Quadriceps	Isokinetic dynamometer. Real-time ultrasound imaging at 28 Hz	Passive peak torque (Nm), MTU stiffness (Nm/°), tendon stiffness (Nm/mm)
Konrad and Tilp (2014a)	*n* = 48 healthy police cadets (IG: *n* = 24. CG: *n* = 24. F = 18. M = 30.), 22.8 ± 2.5 yrs. of age Randomized group allocation High rate of data exclusion due to injuries and poor ultrasound video quality. Post-test: IG: *n* = 20. CG: *n* = 15	Ballistic (dynamic) stretching. Intervention period: 6 wks. Frequency: 5x/wk. Duration per session: 4 × 30s (alternating legs with no rest in-between) Muscle group: Plantar flexors	Isokinetic dynamometer. Real-time B-mode ultrasound (100 mm linear transducer) at 25 Hz	Passive resistive torque (Nm), passive tendon stiffness (N/mm), muscle stiffness (N/mm), MTU stiffness (Nm/°)
Konrad and Tilp (2014b)	*n* = 49 healthy police cadets (IG: *n* = 25. CG: *n* = 24. F = 14. M = 35), 23.1 ± 2.8 yrs. of age Randomized group allocation High rate of data exclusion due to injuries and poor ultrasound video quality. Post-test: IG: *n* = 19. CG: *n* = 15	Static stretching. Intervention period: 6 wks. Frequency: 5x/wk. Duration per session: 4 × 30s (alternating legs with no rest in-between) Muscle group: Plantar flexors	Isokinetic dynamometer. Real-time B-mode ultrasound (100 mm linear transducer) at 25 Hz	Passive resistive torque (Nm), passive tendon stiffness (N/mm), muscle stiffness (N/mm), MTU stiffness (Nm/°)
Konrad et al. (2015)	*n* = 49 healthy police cadets (IG: *n* = 25. CG: *n* = 24. F = 18. M = 31), 23.5 ± 2.7 yrs. of age Randomized group allocation High rate of data exclusion due to injuries and poor ultrasound video quality. Post-test: IG: *n* = 16. CG: *n* = 15	Proprioceptive neuromuscular facilitation (contract-relax-antagonist-contract). Intervention period: 6 weeks. Frequency: 5x/wk. Duration per session: 4 × 15s stretch followed by 6s agonist isometric contraction and 15s antagonist isometric contraction (alternating legs with no rest in-between) Muscle group: Plantar flexors	Isokinetic dynamometer. Real-time B-mode ultrasound (100 mm linear transducer) at 25 Hz	Passive resistive torque (Nm), passive tendon stiffness (N/mm), muscle stiffness (N/mm), MTU stiffness (Nm/°)
Kubo et al. (2002)	*n* = 8 healthy men (intra-individual control leg), 24.6 ± 1.8 yrs. of age. Recreationally active without prior experience in strength and flexibility training programs Randomized allocation of control/intervention leg	Self-administered static stretching. Intervention period: 20 days. Frequency:14x/wk. Duration per session: 5 × 45s with a 15s rest in-between Muscle group: Plantar flexors	Dynamometer. Real-time ultrasound at 30 Hz	Muscle stiffness (N/mm)
LaRoche and Connolly (2006)	*n* = 29 healthy men (IG-SS: *n* = 9. IG-DS: *n* = 10. CG: *n* = 10), 31.6 ± 15.2 yrs. of age. Recreationally active without organized strength and/or flexibility programs in the past 6 months Randomized group allocation	Supervised static and ballistic (dynamic) stretching. Intervention period: 4 wks. Frequency: 3x/wk. Duration per session: 10 × 30s Muscle group: Hamstrings	Isokinetic dynamometer. Electrogoniometer	Passive peak torque (Nm), MTU stiffness (Nm/°)
Longo et al. (2021)	*n* = 30 healthy participants (IG: *n* = 15. CG: *n* = 15. F = 12. M = 18), 22.7 ± 1.8 yrs. of age. Recreationally active university students (aver. weekly training 2 ± 1h) Randomized group allocation	Supervised static stretching. Intervention period: 12 wks. Frequency: 5x/wk. Duration per session: 2 exercises of 5 × 45s with 15s rest in-between Muscle group: Plantar flexors	Custom-made ergometer. B-mode ultrasound (50 mm, 3.1–10.0 MHz linear transducer) in extended-field-of-view mode	Passive resistive peak torque (Nm), MTU stiffness (Nm/°), muscle stiffness (Nm/cm)
Mahieu et al. (2007)	*n* = 96 healthy participants (IG-SS: *n* = 33. IG-DS: *n* = 33. CG: *n* = 30), 22.1 ± 1.6 yrs. of age. Recreationally active Randomized group allocation High dropout-rate due to injury, sickness, or not completing the prescribed program successfully (determined via questionnaire) resulting in n = 81 participants (IG-SS: *n* = 31. IG-DS: *n* = 21. CG: *n* = 29. F = 44. M = 37) for statistical analysis	Self-administered static and ballistic (dynamic) stretching. Intervention period: 6 wks. Frequency: 7x/wk. Duration per session: 5 × 20s with a 20s rest in-between. Dynamic stretching included up-and-down movement of the front, non-stretched knee to change the static to a dynamic stretching Muscle group: Plantar flexors	Isokinetic dynamometer. Real-time ultrasound (7.5 MHz linear transducer)	Passive resistive torque (Nm), tendon stiffness (N/mm)
Mahieu et al. (2009)	*n* = 74 healthy participants (IG: *n* = 37. CG: *n* = 37), 21.9 ± 2.1 yrs. of age. Recreationally active Randomized group allocation High dropout-rate due to not completing the prescribed program successfully (determined via questionnaire) resulting in *n* = 62 participants (IG: *n* = 33. CG: *n* = 29. F = 29. M = 33) for statistical analysis	Self-administered proprioceptive neuromuscular facilitation (contract-relax-antagonist-contract). Intervention period: 6 wks. Frequency: 7x/wk. Duration per session: 5 × 15s stretch followed by 6s agonist isometric contraction and 15s antagonist isometric contraction Muscle group: Plantar flexors	Isokinetic dynamometer. B-mode ultrasound	Passive resistive torque (Nm), tendon stiffness (N/mm)
Marshall et al. (2011)	*n* = 22 healthy participants (IG: *n* = 11. CG: *n* = 11. F = 8. M = 14), 22.7 ± 3.8 yrs. of age Recreationally active and no regular stretching training within the past year Randomized group allocation	Self-administered static stretching (1x/wk supervised). Intervention period: 4 wks. Frequency: 5x/wk. Duration per session: 4 exercises for gluteal and hamstring muscles with 3 × 30s Muscle group: Hamstring	Isokinetic dynamometer	Maximal stiffness (Nm), MTU stiffness (Nm/°)
Moltubakk et al. (2021)	*n* = 30 healthy participants (intra-individual control). Recreationally active university students with no regular stretching training > 10min/wk Randomized (stratified for dominant and non-dominant) allocation of control/intervention leg Post-testing: *N* = 26 (F = 17. M = 9), 22 ± 1.6 yrs. of age	Self-administered static stretching. Intervention period: 24 wks. Frequency: 7x/wk. Duration per session: 4 × 60s Muscle group: Plantar flexors	Isokinetic dynamometer. B-mode ultrasound (50 mm, 5–12 MHz linear transducer) at 38–53 Hz	Passive torque (Nm), tendon stiffness (N/mm)
Nakamura et al. (2012)	*n* = 18 healthy men (IG: *n* = 9. CG: *n* = 9), 21.4 ± 1.7 yrs. of age. Randomized group allocation (No information on sports background.)	Self-administered static stretching. Intervention period: 4 wks. Frequency: 7x/wk. Duration per session: 2 × 60s Muscle group: Plantar flexors	Isokinetic dynamometer. B-mode ultrasound (8 MHz linear transducer)	Passive torque at 0° and 30° (Nm)
Nakamura et al. (2017)	*n* = 24 healthy men (IG: *n* = 12. CG: *n* = 12), 23.8 ± 2.3 yrs. of age. Recreationally active Participants were allocated to IG and CG using the alternation method	Static stretching. Intervention period: 4 wks. Frequency: 3x/wk. Duration per session: 4 × 30s Muscle group: Plantar flexors	Isokinetic dynamometer. B-mode ultrasound (8 MHz linear transducer)	Passive torque (Nm), muscle stiffness (Nm/cm)
Nakamura et al. (2021)	*n* = 40 healthy men (IG-SS-HI: *n* = 14. IG-SS-LI: *n* = 13. CG: *n* = 13), 21.5 ± 1.1 yrs. of age University students not involved in amateur or professional sports Randomized group allocation	High- and low-intensity static stretching. Intervention period: 4 wks. Frequency: 3x/wk. Duration per session: 3 × 60s. IG-SS-HI stretching intensity 6–7 /11, IG-SS-LI stretching intensity 0–1 /11 Muscle group: Plantar flexors	Isokinetic dynamometer. B-mode ultrasound (8 MHz linear transducer) at 56 Hz	Passive torque (Nm), muscle stiffness (N/mm)
Nakamura et al. (2021)	*n* = 15 healthy men (intra-individual control leg), 21.5 ± 1.5 yrs. of age. No competitive athletes and no regular resistance or stretching training No randomization and dominant side as intervention leg	Supervised static stretching. Intervention period: 5 wks. Frequency: 2x/wk. Duration per session: 6 × 300s Muscle group: Plantar flexors	Isokinetic dynamometer. B-mode ultrasound (8 MHz linear transducer)	Passive torque (Nm), muscle stiffness (N/mm)
Peixinho et al. (2021)	*n* = 20 healthy men (IG: *n* = 12. CG: *n* = 8), 18.94 ± 0.45 yrs. of age. Recreationally active but not engaged in regular athletic programs Randomized group allocation	Supervised static stretching. Intervention period: 10 wks. Frequency: 4-5x/wk. Duration per session: 2 exercises each 2 × 30s (alternating legs with no rest in-between) Muscle group: Plantar flexors	Isokinetic dynamometer. B-mode ultrasound (40 mm, 6–11 MHz and 80 mm, 6–11 MHz linear transducers)	Passive peak torque (Nm), mean tangent modulus (MPa), tension deformation (MPa)
Rees et al. (2007)	*n* = 20 healthy women (IG: *n* = 10. CG: *n* = 10), 19.7 ± 1.6 yrs. of age. Physical activity of at least 30 min three times per week Randomized group allocation	Supervised proprioceptive neuromuscular facilitation via contract-relax agonist-contract method in a stretching apparatus. Intervention period: 4 wks. Frequency: 3x/wk. Duration per session: 4–6 sets of 2 repetitions (6–10 s of contraction with 2 s rest in-between repetitions) Muscle group: Plantar flexors	Strain-gage measurement	MTU stiffness (N/m)
Reid and McNair (2004)	*n* = 43 healthy male secondary children (IG: *n* = 23. CG: *n* = 20), 15.8 ± 1 yrs. of age Coin toss to randomly assign the schools to control or intervention	Supervised static stretching. Intervention period: 6 wks. Frequency: 5x/wk. Duration per session: 3 × 30s Muscle group: Hamstrings	Isokinetic dynamometer	Passive resistive torque (N), MTU stiffness (N/°)
Reiner et al. (2023)	*n* = 38 healthy participants (IG: *n* = 19. CG: *n* = 19. F = 15. M = 23), 26.4 ± 5.3 yrs. of age. Physically active (aver. weekly training 9.8 ± 5.5h) Randomized group allocation (stratification male/female)	Self-administered static stretching. Intervention period: 7 wks. Frequency: 3x/wk. Duration per session: 3 × 300s Muscle group: Pectoralis major	Ultrasound shear wave elastography (4–15 MHz linear transducer)	Muscle stiffness at long and short muscle length (kPa)

*IG* Intervention group, *CG* Control group, *n* Number of participants, *MDS* Muscle-directed stretching, *N* Newton, *Nm* Newton meter, *mm* Millimeter, *cm* Centimeter, ° Degree, B-mode = Brightness mode, *NDS* Nerve-directed stretching, *yrs.* Years, *ROM* Range of motion, *MTU* Muscle–tendon-unit, *wks* weeks, *F* Female, *M* Male, *IG-SS-HI* Intervention group with high-intensity static stretching, *IG-SS-LI* Intervention group with low-intensity static stretching

**Table 2:** Study characteristics of the included acute studies

Study	Participants	Stretching	Testing	Outcome
Hatano et al. (2022)	n = 16 healthy participants (cross-over design. F = 8. M = 8), 21.3 ± 0.7 yrs. of age. Recreationally active or sedentary Randomized cross-over sequence	Supervised static stretching. Duration of stretch: 1 × 300s. 3 different IGs with 100%, 110%, 120% stretching intensity Muscle group: Hamstrings	Isokinetic dynamometer	MTU Stiffness (Nm/°), passive torque (Nm)
Herda et al. (2009)	*n* = 15 healthy men (cross-over design), 24 ± 3 yrs. of age. No competitive athletes, moderately-active (6/15: 1–3 h/wk aerobic exercise, 9/15: 2—4 h/wk resistance exercise, 3/15: 1—4 h/wk recreational sport) Randomized cross-over sequence	Supervised static stretching. Duration of stretch: 9 × 135s with 5s rest between bouts Muscle group: Plantar flexors	Isokinetic dynamometer	MTU stiffness
Ikeda et al. (2021)	*n = *10 healthy men (cross-over design), 25 ± 3 yrs. of age Randomized cross-over sequence (No info on sports background.)	Supervised static stretching. Duration of stretch: 5 × 60s with 20s rest intervals Muscle group: Plantar flexors	Isokinetic dynamometer, B-mode ultrasound (60 mm, 7.5 MHz linear transducer) at 32 Hz. Shear wave elastography	Tendon stiffness (N/mm), muscle stiffness (m/s/°)
Iwata et al. (2019)	*n* = 24 healthy participants (IG and CG each n = 12, F = 6, M = 6), 21.4 ± 0.9 yrs. of age. Non-randomized group allocation within a sequential study design	Dynamic stretching. Duration of stretch: 10 × 30s (2s stretch × 15 repetitions) with 20s inter-set rest Muscle group: Hamstrings	Isokinetic dynamometer	Passive torque at the onset of pain (Nm), MTU stiffness (Nm/°)
Kaneda et al. (2020)	*n* = 17 healthy men (cross-over design), 23.2 ± 1.1 yrs. of age. All participants were not involved in competitive sports or regular resistance, aerobic or flexibility training Randomized cross-over sequence	Supervised dynamic stretching. Duration of stretch: 4 × 30s (2s stretch × 15 repetitions) with a 20s rest between bouts Muscle group: Hamstrings	Isokinetic dynamometer	Passive torque (Nm), passive stiffness (Nm/°)
Konrad et al. (2017)	*n* = 122 healthy police cadets (IG-SS: *n* = 25. IG-DS: *n* = 24. IG-PNF: *n* = 49. CG: *n* = 24. F = 43. M = 75), 23.3 ± 2.9 yrs. of age Randomized group allocation Due to poor video quality of ultrasound videos *n* = 25 subjects were excluded resulting in IG-SS: *n* = 22, IG-DS: *n* = 20, IG-PNF: *n* = 31, CG: *n* = 24 for analysis	Supervised static stretching, ballistic (dynamic) stretching, proprioceptive neuromuscular facilitation. Duration of stretch: 4 × 30s for SS and DS, dynamometer applied a passive stretch at the last 5° of the subjects individual ROM (DS), 15s stretch followed by 6s agonist isometric contraction and 15s antagonist isometric contraction (PNF). 20s rest between bouts for all 3 IGs Muscle group: Plantar flexors	Isokinetic dynamometer. Real-time B-mode ultrasound (100 mm linear transducer) at 25 Hz	Passive resistive torque (Nm), passive tendon stiffness (N/mm), muscle stiffness (N/mm), muscle–tendon stiffness (Nm/°)
Konrad et al. (2019)	*n* = 14 healthy participants (cross-over design. F = 7. M = 7), 26.2 ± 5.7 yrs. of age Randomized cross-over sequence (No info on sports background.)	Supervised static stretching. Duration of stretch: 5 × 60s with a 20s rest between bouts Muscle group: Plantar flexors	Isokinetic dynamometer. Real-time B-mode ultrasound (100 mm linear transducer) at 25 Hz	Passive resistive torque (Nm), passive tendon stiffness (N/mm), muscle stiffness (N/mm), MTU stiffness (Nm/°)
Krause et al. (2019)	*n* = 16 healthy participants (cross-over design. F = 6. M = 10), 32.1 ± 5 yrs. of age Randomized cross-over sequence (No info on sports background.)	Supervised static stretching. Duration of stretch: 2 × 60s with a 30s rest between bouts Muscle group: Quadriceps	Isokinetic dynamometer. 3D ultrasound (38.4 mm, 4–11.4 MHz linear transducer) at 10 Hz	Mean passive torque (Nm), mean passive stiffness (Nm/°)
Kuruma et al. (2013)	*n* = 20 healthy participants (F = 20. M = 20. IG: *n* = 10. CG: *n* = 10), 21 yrs. of age (range: 19–24) Randomized group allocation (No info on sports background.)	Static stretching. Duration of stretch: 8 min Muscle group: Quadriceps	Durometer	Muscle stiffness (mN)
Mizuno (2017)	*n* = 15 healthy participants (cross-over design. F = 7. M = 8), 23 ± 2 yrs. of age Randomized cross-over sequence (No info on sports background.)	Supervised dynamic stretching via dynamometer. Duration of stretch: 3 different IGs with 1, 4 and 7 sets of 30s (2s stretch × 15 repetitions) with a 20s rest between bouts Muscle group: Plantar flexors	Isokinetic dynamometer. B-mode ultrasound (45 mm, 12 MHz linear transducer) at 30 Hz	Passive torque (Nm), MTU stiffness (Nm/°)
Mizuno and Umemura (2016)	*n* = 13 healthy participants (cross-over design), 23 ± 1 yrs. of age. Sports students Randomized cross-over sequence Post-test: *N* = 12 (F = 4. M = 8)	Supervised dynamic stretching via dynamometer. Duration of stretch: 4 × 30s (2s stretch × 15 repetitions) with a 20s rest between bouts Muscle group: Plantar flexors	Isokinetic dynamometer. B-mode ultrasound (45 mm, 12 MHz linear transducer) at 30 Hz	Passive torque (Nm), MTU stiffness (Nm/°)
Mizuno (2023)	*n* = 16 healthy participants (cross-over design. F = 9. M = 7), 20.8 ± 0.8 yrs. of age. Participants did not undertake a structured physical training regimen Randomized cross-over sequence	Supervised static stretching and static stretching with additional electrical stimulation. Duration of stretch: 60s Muscle group: Plantar flexors	Isokinetic dynamometer. B-mode ultrasound	Passive torque (Nm), MTU stiffness (Nm/°)
Muir et al. (1999)	*n* = 20 healthy men (intra-individual control), 21–40 yrs. of age Randomized allocation of control/intervention leg (No info on sports background.)	Supervised static stretching. Duration of stretch: 4 × 30s with a 10s rest between bouts Muscle group: Plantar flexors	Isokinetic dynamometer	Passive peak torque (Nm)
Oba et al. (2021)	*n* = 14 healthy men (cross-over design), 22.9 ± 1 yrs. of age Randomized cross-over sequence (No info on sports background.)	Supervised static stretching. Duration of stretch: 5 × 60s with 15s rest between bouts. 3 different IGs at 50%, 75% and 100% of the passive resistive torque Muscle group: Plantar flexors	Isokinetic dynamometer	MTU stiffness (Nm/°)
Opplert and Babault (2019)	*n* = 13 healthy men (cross-over design), 24.9 ± 2.5 yrs. of age. Physically active (aver. weekly training 6.5 ± 2.5h) Randomized cross-over sequence	Supervised static and dynamic stretching. Duration of stretch: 2 × 20s with 20s rest between bouts Muscle group: Plantar flexors	Isokinetic dynamometer	Passive torque at different muscle lengths (Nm)
Palmer et al. (2019)	*n* = 13 healthy women (cross-over design), 21 ± 2 yrs. of age. Physically active (aver. weekly aerobic training 3.9 ± 2.3h and resistance training 5.4 ± 4.2h) Randomized cross-over sequence	Supervised static stretching. Duration of stretch: 3 different IGs with 1 × 30s, 2 × 30s and 4 × 30s Muscle group: Hamstrings	Isokinetic dynamometer	MTU stiffness (Nm/°)
Sonda et al. (2022)	*n* = 32 healthy participants (IG: *n* = 16. CG: *n* = 16), 18–40 yrs. of age. Irregularly active or sedentary Randomized group allocation Post-test: *N* = 30 (IG: *n* = 15. CG: *n* = 15. F = 20. M = 10)	Supervised static stretching. Duration of stretch: 1 × 600s Muscle group: Plantar flexors	Isokinetic dynamometer. B-mode ultrasound (7.5 MHz linear transducer)	Passive torque (Nm), MTU stiffness (Nm/°), muscle stiffness (Nm/cm), tendon stiffness (Nm/cm)
Stafilidis and Tilp (2015)	*N* = 11 healthy participants (cross-over design), 25.5 ± 3.1 yrs. of age. Recreationally active in various sports with approx. activity time of 7h/wk Randomized cross-over sequence	Static stretching. Duration of stretch: 2 different protocols with 1 × 15s and 1 × 60s per muscle group in both legs with no rest in-between Muscle groups: Quadriceps, hamstrings and plantar flexors	Isokinetic dynamometer. B-mode ultrasound (100 mm, 10 MHz linear transducer) at 35 Hz	Passive torque (Nm), MTU stiffness (Nm/cm)
Vieira et al. (2021)	*n* = 13 healthy men (cross-over design), 24.9 ± 2.5 yrs. of age. Physically active (aver. weekly training 6.5 ± 2.5h) Randomized cross-over sequence	Supervised dynamic stretching, static stretching and passive cyclic stretching. Duration per stretch: 2 × 20s with a 20s rest between bouts Muscle group: Plantar flexors	Isokinetic dynamometer. B-mode ultrasound (7.5 MHz linear transducer) at 100 Hz	Passive torque (Nm), fascicle stiffness index (Nm/cm)

*IG* intervention group, *CG* control group, *n* number of participants, *M* male, *F* female, *h* hour(s), *wk* week, *yrs*. years, *SS* static stretching, *DS* dynamic stretching, *s* seconds, *B-mode* brightness mode

Acute-stiffness adaptations were evaluated in 12 studies, while muscle stiffness was tested in eight, and four studies measured tendon stiffness. Six studies evaluated MTU stiffness. PPT in end ROM was tested in only four studies, while measurement at a fixed angle was used in 11 studies.

### Methodological quality, risk of bias, and certainty of the evidence

The methodological quality of the included studies was rated as fair (PEDro score of 4.5 ± 1.1 out of 10 points; range 3 to 8 points). Almost all studies (45 out of 49) used random-group allocation and reported statistical between-group comparisons (44 out of 49), and all provided both point measures and measures of variability (49 out of 49). Blinding of the participants as well as of all the therapists administering the intervention was never reported, while assessors were blinded in 2 out of 49 studies. Very few studies (4 out of 49) declared application of the intention-to-treat principle (see Table A in the supplemental material).

The absence of publication bias was ensured by visual inspection of funnel plots and by performing Egger’s regression tests for the chronic passive torque measurements (*p* = 0.37, 95% CI  −  1.90 to 4.83) and for the overall stiffness assessment (including muscle, tendon, and muscle–tendon stiffness parameters) (*p* = 0.41, 95% CI  −  1.23 to 2.92). Results of the passive torque measurement in the acute effect studies were *p* = 0.35, 95% CI  −  4.37 to 1.69 and the results for the overall stiffness assessment were *p* = 0.95, 95% CI  −  3.07 to 2.90. Funnel plots for chronic stiffness and chronic peak torque in end ROM are provided in Figs. 1 and 2 in the supplemental material.

**Fig. 2:**
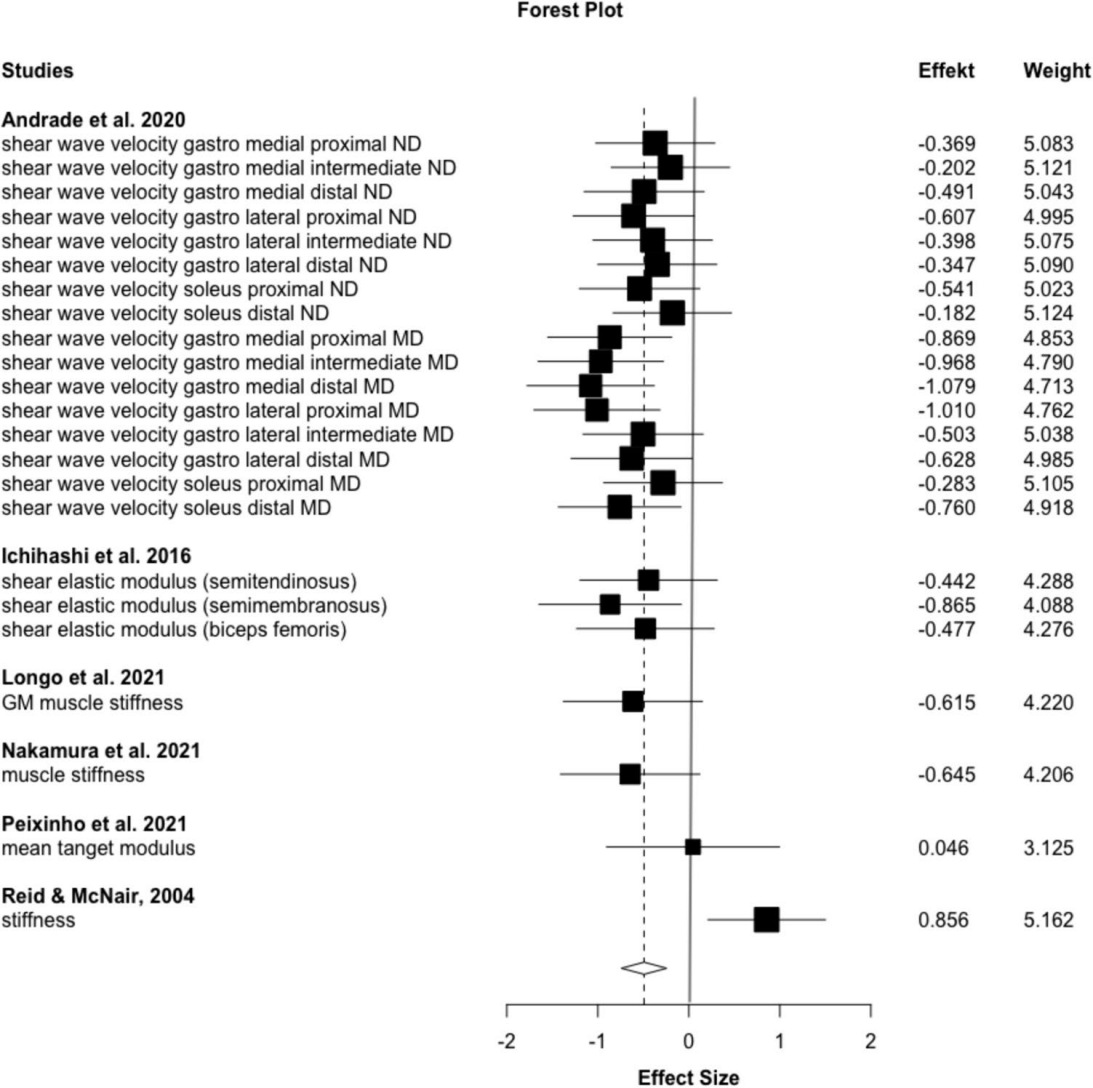
Forest plot for chronic supervised stretching effects on muscle stiffness (excluding the ND subgroup from Andrade still results in an ES:  – 0.55 ( – 0.996 to  – 0.11), *p* = 0.02, from 6 studies and 15 outcomes), *MD* muscle directed, *ND* nerve directed

### Quantitative synthesis

#### Chronic effects of stretching on passive properties

The level of significance was not reached for the overall stiffness (all types, meaning muscle, tendon, and MTU stiffness) (ES =   −  0.12 to  −  0.24, *p* = 0.12–0.56) and for the tendon and MTU-stiffness subgroups (ES =   −  0.07–0.19, *p* = 0.52–0.81). Muscle stiffness, exclusively, was affected by stretching (ES =   −  0.38, *p* = 0.01), with static stretching showing somewhat higher small magnitude effects (ES =   −  0.44, *p* = 0.02). Dynamic and PNF stretching resulted in non-significant results. Only if stretching was supervised (Fig. 2) can small to moderate magnitude effects be found (ES =   −  0.49, *p* = 0.02). Long-stretching durations resulted in moderate-sized effects (ES =   −  0.61, *p* < 0.001), while short duration effects did not reach the level of significance (*p* = 0.21). PPT in end ROM was only measured after static interventions, showing a moderate magnitude increase (ES = 0.55, *p* = 0.005), without being significantly moderated by supervision (*p* = 0.07–0.32) or stretching duration (*p* = 0.05–0.09). No changes were observed when measured in fixed joint-angle positions (Table 3).

**Table 3:** Meta-analytic results for chronic effects of stretching on muscle-, tendon-, and muscle tendon unit stiffness and passive torque for overall, static and dynamic/PNF stretching providing effect size, 95% CI, significance, and heterogeneity

Parameter	ES (95% CI)	*p*-value	Heterogeneity	Studies/Outcomes
Chronic stiffness overall	– 0.24 (– 0.56 to – 0.07)	0.12	0.14	18/48
Supervised	– 0.41 (– 0.74 to – 0.08)	0.02*	0.16	8/27
Unsupervised	– 0.03 (– 0.32 to 0.25)	0.8	0.07	12/21
Chronic stiffness overall static	– 0.29 (– 0.66 to 0.08)	0.12	0.20	15/32
Short (overall duration ≤ 225 s)	– 0.25 (– 0.77 to 0.28)	0.32	0.25	12/26
Long (overall duration > 225 s)	– 0.61 (– 0.69 to -0.54)	< 0.001*	0	3/6
Chronic stiffness overall dynamic	– 0.12 (– 0.60 to 0.36)	0.56	0.06	6/17
Chronic muscle stiffness	– 0.38 (– 0.65 to -0.11)	0.01*	0.12	14/34
Supervised	– 0.49 (– 0.74 to -0.24)	0.004*	0.06	6/23
Unsupervised	– 0.19 (– 0.58 to 0.2)	0.29	0.13	8/11
Chronic muscle stiffness static	– 0.44 (– 0.81 to -0.07)	0.02*	0.18	11/23
Short (overall duration ≤ 225 s)	– 0.36 (– 0.97 to 0.26)	0.21	0.37	8/17
Long (overall duration > 225 s)	– 0.61 (– 0.69 to -0.54)	< 0.001*	0	3/6
Chronic muscle stiffness dynamic	– 0.25 (– 0.71 to 0.20)	0.18	0	4/11
Chronic tendon stiffness	0.04 (– 0.39 to 0.48)	0.81	0.09	7/8
Chronic tendon stiffness static	0.19 (– 0.64 to 1.03)	0.52	0.14	4/4
Chronic tendon stiffness dynamic	– 0.07 (– 0.72 to 0.58)	0.75	0.08	4/4
Chronic PPT end ROM (all static)	0.55 (0.21 to 0.90)	0.005*	0.12	10/23
Short (overall duration ≤ 150 s)	0.66 (– 0.16 to 1.48)	0.09	0.23	5/14
Long (overall duration > 150 s)	0.46 (– 0.007 to 0.92)	0.05	0.05	6/9
Chronic PPT fix angle	– 0.22 (– 0.50 to 0.06)	0.11	0.20	11/23
Chronic PPT fix angle static	– 0.34 (– 0.76 to 0.73)	0.09	0.35	8/17
Short (overall duration < 225 s)	– 0.34 (– 0.76 to 0.08)	0.09	0.79	5/7
Long (overall duration ≥ 225 s)	– 0.35 (– 1.21 to 0.523)	0.29	0.15	4/10
Chronic PPT fix angle dynamic	– 0.02 (– 0.20 to 0.17)	0.83	0	5/6

*Significant effects with *p* < 0.05

#### Acute effects of stretching on passive properties

There were trivial reductions in overall stiffness (ES =   −  0.17, *p* = 0.02) when including all types of stretching. Muscle stiffness was acutely reduced if stretching was performed for long durations (ES =  − 0.37, *p* = 0.02), while other subgroups did not affect stiffness parameters. (Table 4).

**Table 4:** Meta-analytic results for acute effects of stretching on muscle-, tendon-, and muscle tendon unit stiffness and passive torque for overall, static and dynamic/PNF stretching providing effect size, 95% CI, significance, and heterogeneity

Parameter	ES (95% CI)	*p* value	Heterogeneity	Studies/outcomes
Acute-stiffness overall	– 0.17 (– 0.31 to – 0.03)	0.02*	0	12/48
Acute-stiffness overall static	– 0.25 (– 0.47 to – 0.07)	0.015*	0	9/25
Short (overall duration ≤ 300 s)	– 0.33 (– 0.56 to – 0.10)	0.014*	0	6/17
Long (overall duration > 300 s)	– 0.34 (– 1.79 to 1.1)	0.20	0	2/4
Acute-stiffness overall dynamic	– 0.08 (– 0.31 to 0.15)	0.40	0	5/24
Acute muscle stiffness	– 0.27 (– 0.58 to 0.04)	0.08	0	8/15
Acute muscle stiffness static	– 0.21 (– 0.56 to 0.13)	0.18	0	7/12
Short (overall duration < 300 s)	– 0.04 (– 2.22 to 2.14)	0.84	0	2/5
Long (overall duration ≥ 300 s)	– 0.37 (– 0.64 to – 0.11)	0.02*	0	5/7
Acute muscle stiffness dynamic	– 0.37 (– 1.23 to 0.50	0.21	0	3/4
Acute tendon stiffness	– 0.45 (– 1.16 to 0.26)	0.14	0.04	4/4
Acute MTU stiffness	– 0.07 (– 0.22 to 0.08)	0.27	0	6/25
Acute MTU stiffness static	– 0.23 (– 0.5 to 0.03)	0.07	0	4/9
Acute MTU stiffness dynamic	0.02 (– 0.49 to 0.53)	0.66	0	2/16
Acute PPT end ROM	0.54 (– 0.31 to 1.39)	0.14	0.27	4/6
Acute PPT fix angle	– 0.05 (– 0.18 to 0.09)	0.45	0	11/51
Acute PPT fix angle static	– 0.05 (– 0.29 to 0.18)	0.61	0	10/24
Short (overall duration < 120 s)	– 0.23 (– 0.58 to 0.13)	0.11	0	3/8
Long (overall duration ≥ 120 s)	0.007 (– 0.27 to 0.28)	0.95	0	8/16
Acute PPT fix angle dynamic	– 0.04 (– 0.23 to 0.15)	0.59	0	5/29

*Significant effects with *p* < 0.05

#### Certainty about the evidence

Following the rating criteria, the quality of the evidence was initially classified as high as almost all included studies were randomized controlled trials (45 randomized and 4 non-randomized controlled trials). The quality of evidence was downgraded by one level (high to moderate) for unclear risk of bias (limitations in study quality: fair PEDro score). For the results of the acute effect studies, the quality of evidence was further downgraded by one level (moderate to low) for imprecision of the ES. On the contrary, the quality of evidence for the chronic effect studies was upgraded by one level for the large effect magnitude. In summary, while the quality of evidence is high for the chronic effect studies, it is low for the acute ones.

## Discussion

The presented review evaluated the acute and chronic effects of different stretching types on passive muscle properties and PPT. In accordance with the previous literature reporting neuromuscular adaptations as underlying mechanisms for ROM enhancements (Freitas et al. 2018), our results showed moderate magnitude chronic PPT effects measured in end ROM. Furthermore, the results partially agree with those provided by Takeuchi et al. (2023a), as only static stretching reduced stiffness acutely and chronically, favoring long-stretching durations and supervised training.

While previous analyses focused on the acute or chronic effects of either muscle, MTU, or tendon stiffness without investigating passive torque parameters (Takeuchi et al. 2023a, b), there were partially conflicting results, compared to the Freitas et al. (2018) review. Furthermore, it must be noted that both of the 2023 reviews by Takeuchi et al. did not use multilevel approaches. Consequently, the differences between the results of this meta-analysis and previous analyses could result from the inclusion of a larger number of studies (Freitas et al. 2018) and the different calculation models (Freitas et al. 2018; Takeuchi et al. 2023a, b). Simple statistical approaches pool ES considering each included effect as independent and equally weighted; however, they neglect the dependency of multiple outcome measures (Fisher and Tipton 2015). Therefore, meta-analytical approaches should account for effect size dependency by more than averaging multiple outcomes (Becker 2000). Using the RVE model is therefore suggested as being the most appropriate procedure for valid standard error determination, point estimates, CIs, and significance tests (Fisher and Tipton 2015) when including multiple study outcomes with heteroscedastic data structures, which is commonly assumed in sport and exercise science research (Atkinson and Nevill 1998; Hopkins 2000). Therefore, to the best of our knowledge, this review reduced the methodological bias sufficiently, while extending the current knowledge of underlying stretching mechanisms, among others, by adding PPT results.

### Muscle stiffness

Reiner et al. (2024) classified the correlation coefficients of  −  0.43 to  −  0.5 between muscle stiffness in the ischiocrural muscles and hip flexion ROM as moderate to large, and suggested stiffness as being an important contributor for flexibility. However, on the one hand, correlations do not suggest a causal relationship; on the other hand, flexibility must be considered a highly complex construct, influenced by several factors of neural and morphologic nature, underlining correlations between 0.43 and 0.50 as potentially meaningful. However, even though logical, it is necessary to investigate the association in longitudinal research designs.

Muscle stiffness is commonly measured via ultrasound elastography, either strain or shear wave. Within this study, to counteract the common limitations regarding method heterogeneity in sport and exercise meta-analytical approaches, we focused on shear wave elastography only, as it is mostly performed. While providing an overall effect is methodologically biased by the different data collection procedures (dynamometer for MTU, shear wave elastography for muscle or tendon stiffness), subgroup analyses for each stiffness parameter were performed to further decrease the method heterogeneity. The subgroup analysis for muscle stiffness was affected by stretching in general (ES:  −  0.38 *p* = 0.01), while the effectivity might be attributable to static stretching (overall ES:  −  0.44, *p* = 0.02), favoring long-stretching durations (ES:  −  0.61, *p* < 0.001). Further subgroups did not reach the level of significance. Although of smaller magnitude, these results are in accordance with those of Takeuchi et al. (2023a, b). Since there was a huge overlap in the included studies, these differences could be due to the different effect size calculation methods. The lack of significant results in the Freitas et al. (2018) study could be additionally attributed to the additional research published after 2018.

Especially when aiming to affect the structural parameters of the muscle via stretching, the literature underlines the relevance of intensity (Apostolopoulos et al. 2015; Panidi et al. 2023). While the quantification of stretching intensity via subjective pain feeling seems biased, it is known from other research areas that supervision can influence the effectivity of training routines (Lacroix et al. 2017). Especially since stretching above the pain threshold can be uncomfortable for the participant, but important for the magnitude of the effects (Panidi et al. 2023), we checked whether supervision affected the underlying parameters. Accordingly, supervision of the stretching sessions seems to moderate ES. When comparing non-supervised sessions to supervised, only the supervised subgroup showed significant muscle stiffness changes. Even though only indirectly indicated, supervision could have caused more appropriate exercise execution, and thus more pronounced effects. However, investigations that explored direct comparisons of supervision vs. non-supervision on stiffness were not found. Only for balance performance in resistance training programs did Lacroix et al. (2017) point out the higher effectivity of supervised conditions as well. However, the origin of stiffness decreases still remains speculative. One possible explanation could be a structural elongation of the muscle, however, in humans, researchers have only speculated about stretch-induced sarcomerogenesis (Kruse et al. 2021; Zöllner et al. 2012), while there is a large body of evidence reporting sarcomerogenesis in animal research, especially in response to long-stretching interventions (Kelley 1996; Warneke et al. 2022a).

Similarly, acute effects showed static stretching to decrease the overall stiffness (ES:  −  0.25, *p* = 0.015), while muscle stiffness was only reduced in response to long-stretching durations (ES:  −  0.37, *p* = 0.02). The remaining subgroup analyses did not surpass the α = 0.05 threshold and remained insignificant. Especially for the tendon and muscle stiffness subgroups, only a small study sample with comparatively limited possibilities to pool ES could be included in the analytical calculation, calling for high-quality stretching studies including a passive control condition. The underlying mechanism of acutely decreased muscle stiffness could be, on the one hand, attributed to thixotropic effects; however, a recently published meta-analysis indicated stiffness decreased acutely, independent of using stretching or alternative interventions (Warneke et al. 2024a, b, c). These results could indicate that stretching, among other techniques, results in increased muscle temperature (Oliveira et al. 2018), which could reduce friction, induce thixotropy, and thus improve viscoelastic properties, which would cause reduced stiffness. Nevertheless, stretch-related parameters that change muscle stiffness should be studied in further research.

### Tendon stiffness

No stretching effects could be found on tendon stiffness. Since only 4 studies were available for static stretching, no subgroup analysis for stretching duration was performed. Also stretching type did not significantly influence this result. However, since also for tendon stiffness investigations very long-stretching durations sufficient to change structural properties (Panidi et al. 2023; Warneke et al. 2024a, b, c) are still missing, there is a demand of very long duration stretches to draw valid conclusions on acute and chronic stretching effects on tendon stiffness.

### Passive torque

When exploring PPT, the location of the measurement influences the results. On the one hand, measuring PPT in end ROM is often equated with a later occurrence of stretching pain (Folpp et al. 2006). On the other hand, determining the PPT in a fixed ROM position can be considered more related to structural parameters, showing reduced resistance against an external force in a predetermined joint angle. Surprisingly, even though moderate-sized acute PPT enhancements were revealed in end ROM with ES = 0.54, the pooled effect size failed to reach the level of significance. Similar to the stiffness subgroups, the resulting sample size was based on six ES extracted from four studies, which clearly affected the level of significance. More studies have addressed passive torque adaptations, leading to the impression of strongly examined stretching-induced acute PPT effects, but these studies were performed without control conditions, and thus were excluded from this review. As we focused on controlled studies only, a large amount of literature which used uncontrolled conditions, mathematical models, or compared different types of stretching/intensities/volumes without including a control group had to be excluded from this review (Chen et al. 2018; Fukaya et al. 2021; Matsuo et al. 2023; McNair & Stanley 1996; Nordez et al. 2006, 2009a, b; Reiner et al. 2022). Consequently, this substantial lack in literature calls for future research.

While no acute change in peak torque could be found, in accordance with previous literature (Freitas et al. 2018; Freitas and Mil-Homens 2015; Konrad and Tilp 2014), there was a moderate effect size for chronic PPT enhancements measured in end ROM (ES: 0.55, *p* = 0.005). These results support previous beliefs of the delayed occurrence in stretch sensation. Nevertheless, when interpreting these results, it must be considered that participants determine the position of pain occurrence, at which the passive resistance is determined. Since several weeks commonly lie between the pre-test and post-test, and participants within the testing were not blinded, the role of placebo effects due to expected increases in this highly subjective and participant-dependent testing should be considered. Research in which participants are blinded for the movement of the foot is therefore required to validate these effects.

Twenty-three (23) ES from 11 studies measured PPT in a standardized joint angle in the pre-test and post-test, without reaching the level of significance, which argues against structural stretching responses. In general, for PPT (end ROM as well as standardized joint-angle measurement), supervision and stretching time did not moderate these adaptations.

### Can stiffness and peak torque adaptations be reviewed separately?

**Fig. 3:**
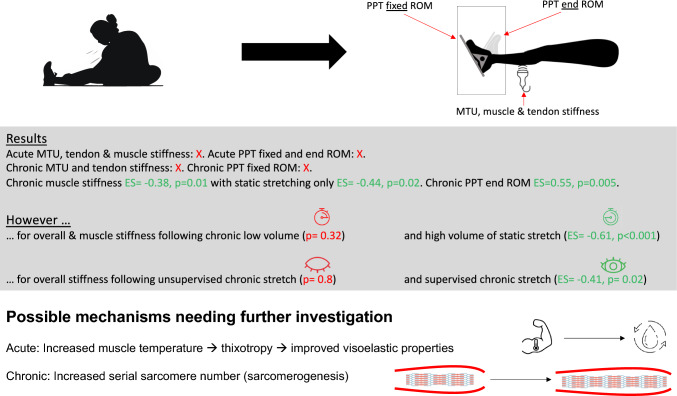
Graphical illustration of the results and underlying mechanisms

Stiffness adaptations, as well as PPT, are often described as the underlying physiologic parameters of flexibility enhancements (Freitas et al. 2015, 2018; Konrad and Tilp 2014). However, even though contributing to flexibility, both seem to be of a more phenomenological nature. Increases of PPT in end ROM can be attributed to more neuronal or psychologic adaptations (pain occurs in higher muscle lengths)(Folpp et al. 2006), which was attributed to a reduced sensibility (Freitas et al. 2018) of nociceptive nerve endings (Julius and Basbaum 2001; Støve et al. 2021), while stiffness decreases would indicate morphologic adaptations. Considering pain as a warning signal prior to possibly harming oneself and causing pathologies or injury (Julius and Basbaum 2001; Sirianni et al. 2015; Støve et al. 2021), it could be speculated that, in end ROM, this could lead to not being able to exert a higher force, with a potential negative implication for joint stability in high muscle lengths. Rassier et al. (1999) described the active force output to be maximal when the filament overlap is maximal as well, while a decreased overlap would diminish the exerted muscle force. Assuming that sarcomere and muscle stretch causes a reduced overlap of contractile filaments, stretching pain and increased passive resistance of the muscle could potentially be described as warning signals for limited joint stability in high muscle lengths. Assuming longitudinal hypertrophy (increased serial sarcomere number) would allow the muscle to cover higher muscle lengths, which might stabilize the joint and cause a later onset of pain (increased PPT in end ROM). This would, on the one hand, explain the chronic decreases in muscle stiffness and, on the other hand, the higher PPT in end ROM. However, since serial sarcomere adaptation measurements seem to be of limited accessibility, stretch-induced serial sarcomere number adaptations remain elusive, especially as the common length-dependent contraction models are challenged (MacIntosh 2017). The overall results are summarized in Fig. 3.

### Limitations

In this study, meta-analytical calculations were initially used to pool the effects from very homogeneous studies to reach a higher sample size and overall effect from studies with equal or at least similar study designs (Borenstein et al. 2009). Even though the random effects model and RVE pooling were performed, between-study variations arising from different study designs bias the results of meta-analyses in general. Therefore, several subgroup analyses for moderators extracted from the literature were performed to enhance the comparability of the effects. Furthermore, some effects collected in studies using incomparable methods (e.g., strain elastography) were excluded. To be restrictive, on the one hand, enhances the homogeneity in the results but, on the other hand, excludes further effects that might not include all the available results to reflect the current state of evidence. Therefore, in general, meta-analytical results should be handled with care, as study selection due to eligibility criteria with the resulting sample size, as well as the chosen statistical model, can affect the results.

Surprisingly, only four acute stretching studies including PPT measured in end ROM with a controlled study design could be included. This sample size strongly limits the interpretability of the effect size pooling and calls for further research.

Furthermore, the subgroup median split was calculated for each individual subgroup to divide the study number and oppose small stretching durations with the longer duration study effects. This led to different cut offs, which prevents direct comparisons and overall stretch duration recommendations, thus small and long classifications are partly based on different stretching times, depending on the used interventions in literature.

### Practical applications

In (partial) accordance with previous reviews, the presented results indicate stiffness and passive torque adaptations after performing chronic stretching interventions for several weeks, while our analysis showed acute-stiffness reductions, without the subgroups reaching the level of significance. Therefore, higher stretching durations under supervision, especially, seem to induce (chronic) stiffness reductions accompanied by a delayed occurrence of stretching pain in high muscle lengths. However, the origin of stretching pain remains speculative (neural or structural or combined) and should be investigated in future research. Therefore, when aiming to investigate stretching effects in future research, reasonably long-stretching durations should be induced if morphologic changes are to be investigated. Furthermore, our results strongly suggest that more effort in study design development and intervention conduction is required, as supervision of long intervention periods with extended stretching durations must be supervised to ensure the investigation effectivity in chronic research.

## Supplementary Information

Below is the link to the electronic supplementary material.

Supplementary file1 (DOCX 134 KB)

## Data Availability

Original data (excel lists with extracted data) can be provided by the corresponding author due to reasonable request.

## Abbreviations

M: Mean
MTU: Muscle tendon unit
PNF: Proprioceptive neuromuscular facilitation
PPT: Passive peak torque
ROM: Range of motion
RVE: Robust variance estimation
SD: Standard deviation
SMD: Standardized mean difference
