# Supplementary material for: Critical evaluation and recalculation of current systematic reviews with meta-analysis on the effects of acute and chronic stretching on passive properties and passive peak torque
Source: Eur J Appl Physiol. 2024 Jul 27;124(11):3153–73. doi: 10.1007/s00421-024-05564-6 (PMC11519181; doi:10.1007/s00421-024-05564-6)
Supplement: Supplementary file 1 — Supplementary file1 (DOCX 134 KB) [file 421_2024_5564_MOESM1_ESM.docx]

SUPPLEMENTAL MATERIAL

**Tab. 1: Quality assessment using the PEDro Scale**

| Name of study | 2. | 3. | 4. | 5. | 6. | 7. | 8. | 9. | 10. | 11. | rating |
| --- | --- | --- | --- | --- | --- | --- | --- | --- | --- | --- | --- |
| Akagi & Takahashi, 2013 | Y | N | Y | N | N | N | N | N | Y | Y | 4/10 |
| Andrade et al., 2020 | Y | Y | Y | N | N | N | Y | Y | Y | Y | 7/10 |
| Ben & Harvey, 2010 | Y | Y | Y | N | N | Y | Y | Y | Y | Y | 8/10 |
| Blazevich et al., 2014 | Y | Y | Y | N | N | N | Y | N | Y | Y | 6/10 |
| Brusco et al., 2019 | N | N | Y | N | N | N | N | N | Y | Y | 3/10 |
| Chan et al., 2001 | Y | N | Y | N | N | N | Y | N | Y | Y | 5/10 |
| Cini et al., 2022 | Y | Y | Y | N | N | Y | Y | Y | Y | Y | 8/10 |
| Gajdosik et al., 2005 | Y | Y | Y | N | N | N | N | N | Y | Y | 5/10 |
| Gajdosik et al., 2007 | Y | N | Y | N | N | N | Y | N | Y | Y | 5/10 |
| Halbertsma & Göeken, 1994 | Y | N | Y | N | N | N | Y | N | N | Y | 4/10 |
| Hatano et al., 2022 (Acute) | Y | N | Y | N | N | N | N | N | Y | Y | 4/10 |
| Herda et al., 2009 (Acute) | Y | N | Y | N | N | N | Y | N | Y | Y | 5/10 |
| Ichihashi et al., 2016 | Y | N | Y | N | N | N | Y | N | Y | Y | 6/10 |
| Ikeda et al., 2020 (Acute) | Y | N | Y | N | N | N | N | N | Y | Y | 4/10 |
| Iwata et al., 2019 | N | N | Y | N | N | N | Y | N | Y | Y | 4/10 |
| Kaneda et al., 2020 (Acute) | Y | N | Y | N | N | N | N | N | Y | Y | 4/10 |
| Kay et al., 2018 | Y | N | Y | N | N | N | Y | N | Y | Y | 5/10 |
| Konrad & Tilp, 2014a | Y | N | Y | N | N | N | N | N | N | Y | 3/10 |
| Konrad & Tilp, 2014b | Y | N | Y | N | N | N | N | N | N | Y | 3/10 |
| Konrad et al., 2015 | Y | N | Y | N | N | N | N | N | N | Y | 3/10 |
| Konrad et al., 2017 (Acute) | Y | N | Y | N | N | N | N | N | N | Y | 3/10 |
| Konrad et al., 2019 (Acute) | Y | N | Y | N | N | N | N | N | Y | Y | 4/10 |
| Krause et al., 2019 (Acute) | Y | N | Y | N | N | N | N | Y | Y | Y | 6/10 |
| Kubo et al., 2002 | Y | N | Y | N | N | N | N | N | Y | Y | 4/10 |
| Kuruma et al., 2013 (Acute) | Y | N | Y | N | N | N | N | N | Y | Y | 4/10 |
| LaRoche & Connolly, 2006 | Y | N | Y | N | N | N | Y | N | Y | Y | 5/10 |
| Longo et al., 2021 | Y | N | Y | N | N | N | Y | N | Y | Y | 6/10 |
| Mahieu et al., 2007 | Y | Y | Y | N | N | N | N | N | Y | Y | 5/10 |
| Mahieu et al., 2009 | Y | Y | Y | N | N | N | N | N | Y | Y | 5/10 |
| Marshall et al., 2011 | Y | N | Y | N | N | N | Y | N | Y | Y | 5/10 |
| Mizuno, 2016 (Acute) | Y | N | Y | N | N | N | N | N | Y | Y | 4/10 |
| Mizuno & Umemura, 2016 (Acute) | Y | N | Y | N | N | N | Y | N | Y | Y | 5/10 |
| Mizuno, 2023 (Acute) | Y | N | Y | N | N | N | N | N | Y | Y | 4/10 |
| Moltubakk et al., 2021 | Y | N | Y | N | N | N | Y | N | Y | Y | 5/10 |
| Muir et al., 1999 (Acute) | Y | N | Y | N | N | N | N | N | Y | Y | 4/10 |
| Nakamura et al., 2012 | Y | N | Y | N | N | N | N | N | Y | Y | 4/10 |
| Nakamura et al., 2016 | N | N | Y | N | N | N | N | N | Y | Y | 3/10 |
| Nakamura et al., 2021a | Y | N | Y | N | N | N | N | N | Y | Y | 4/10 |
| Nakamura et al., 2021b | N | N | Y | N | N | N | N | N | Y | Y | 3/10 |
| Oba et al., 2021 (Acute) | Y | N | Y | N | N | N | N | N | Y | Y | 4/10 |
| Opplert & Babault, 2019 (Acute) | Y | N | Y | N | N | N | N | N | Y | Y | 4/10 |
| Palmer et al., 2019 (Acute) | Y | N | Y | N | N | N | N | N | Y | Y | 4/10 |
| Peixinho et al., 2021 | Y | N | Y | N | N | N | N | N | Y | Y | 4/10 |
| Rees et al., 2007 | Y | N | Y | N | N | N | Y | N | Y | Y | 5/10 |
| Reid & McNair, 2004 | Y | N | Y | N | N | N | N | N | Y | Y | 5/10 |
| Reiner et al., 2023 | Y | Y | Y | N | N | N | N | N | Y | Y | 5/10 |
| Sonda et al., 2022 (Acute) | Y | N | Y | N | N | N | N | N | Y | Y | 4/10 |
| Stafilidis et al., 2015 (Acute) | Y | N | Y | N | N | N | N | N | Y | Y | 4/10 |
| Vieira et al., 2021 (Acute) | Y | N | Y | N | N | N | N | N | Y | Y | 4/10 |
| Mean | | | | | | | | | | | 4.5/10 |


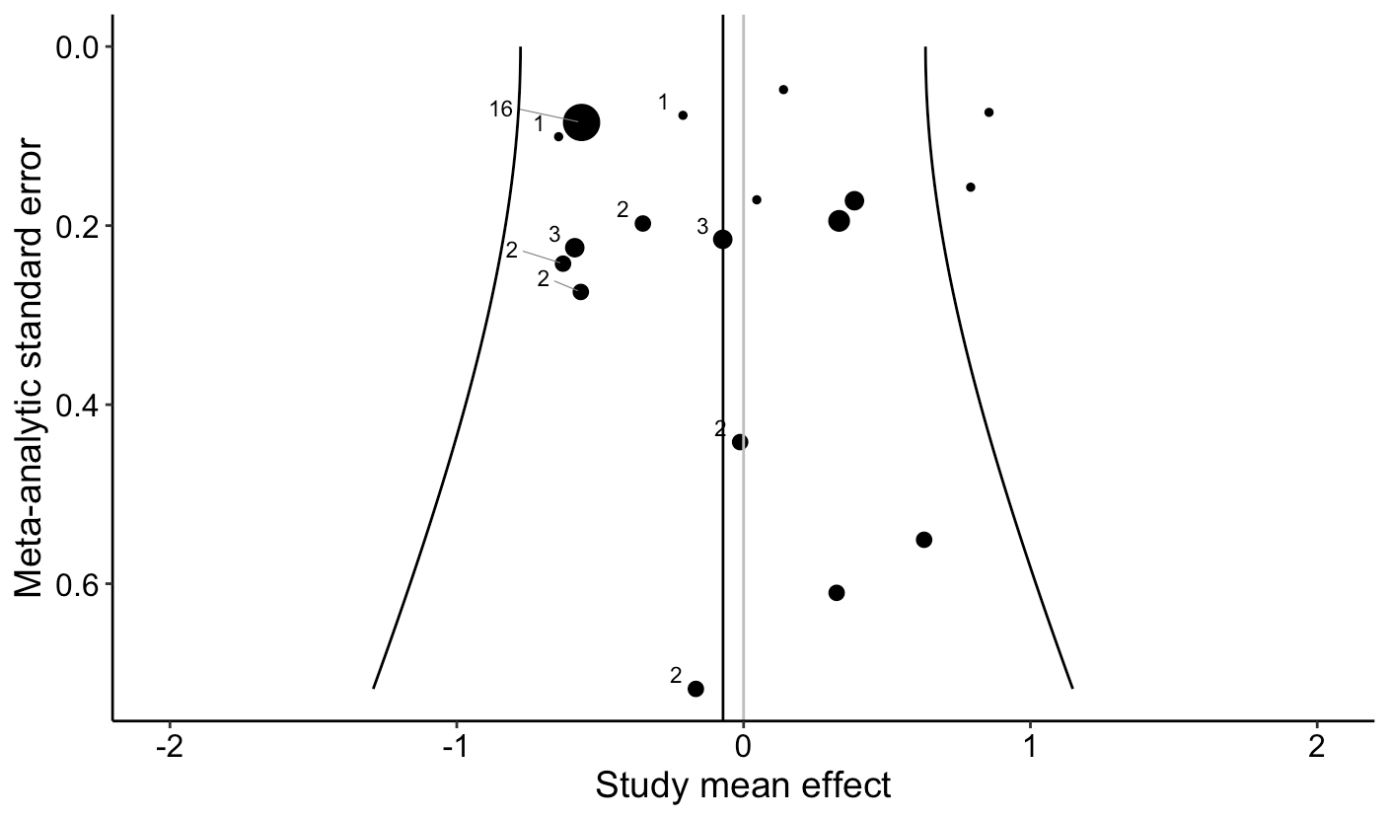


**Fig. 1** Funnel plot for chronic stiffness adaptations


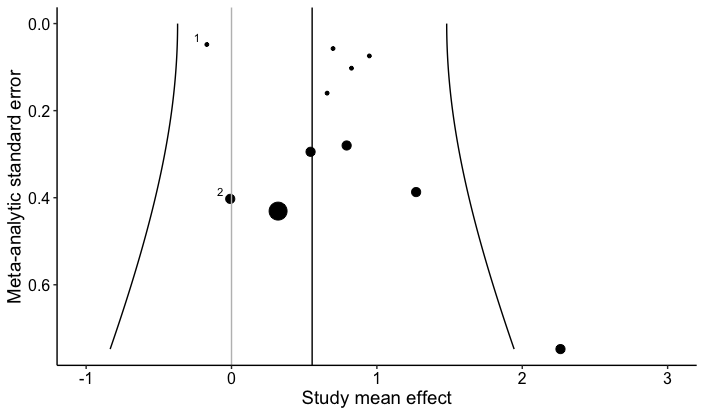


**Fig. 2** Funnel plot for chronic passive peak torque in end ROM adaptations
